# Alcohol use during pregnancy and associated factors among pregnant women in Sub-Saharan Africa: further analysis of the recent demographic and health survey data

**DOI:** 10.1186/s12884-022-04694-z

**Published:** 2022-04-26

**Authors:** Bezawit Mulat, Wallelign Alemnew, Kegnie Shitu

**Affiliations:** 1 Department of Human Physiology, School of Medicine, College of Medicine and Health Sciences, University of Gondar, Gondar, Ethiopia; 2 Department of Health Education and Behavioral Sciences, Institute of Public Health College of Medicine and Health Sciences, University of Gondar, Gondar, Ethiopia

**Keywords:** Alcohol drinking, Pregnancy, Sub-Saharan Africa

## Abstract

**Background:**

Alcohol drinking during pregnancy is towering despite the well-established proof of its unfavorable pregnancy results and destitute child improvement. Despite such enormous consequences, there are limited data that explore the extent of alcohol drinking and its associated factors among mothers during pregnancy in sub-Saharan Africa.

**Objective:**

This study aimed to assess the prevalence and associated factors of alcohol consumption during pregnancy among pregnant mothers in sub-Saharan Africa.

**Method:**

A community-based crossectional demographic and health survey was conducted from 2013 to 2017 among four Sub-Sahara African countries: Burundi, Ethiopia, Liberia, and Zimbabwe. A two-stage stratified sampling technique was employed to select the participants. Multivariable Logistic regression analysis was used to identify factors associated with alcohol consumption during pregnancy. A *p*-value less than 0.05 and a 95% confidence interval were used to declare statistical significance.

**Result:**

A total of 3953 weighted sample of pregnant mothers were included in the study. The mean age of the participants was 27.3 (± 6.8) years with an age range of 15–49 years. The overall prevalence of alcohol use during pregnancy was 22.8% with (95% CI (21.5, 24)) and it was significantly associated with increased age (AOR = 1.02, 95% CI (1.01, 1.04)), Muslim religion follower ( AOR = 0.07, 95% CI (0.05,0.11), husband/partner’s educational status( primary (AOR = 0.7,95% CI (0.55,0.84), secondary (AOR = 0.53, 95% CI ( 0.41,0.7)) and higher (AOR = 0.49, 95% CI (0.31,0.8), being currently working (AOR = 1.5,95% CI ( 1.09,1.55), having ANC visit ( AOR = 0.82, 95% C I(0.68,0.98) and increased gravidity ( AOR = 0.93,95% CI( 0.86,0.99).

**Conclusion:**

Alcohol drinking during pregnancy was high among pregnant women in sub-Saharan African countries. Maternal age, religion, husband educational status, current working status of the mother, presence of ANC visit, and gravidity of the mother were factors that have a significant association with alcohol drinking during pregnancy. This calls for a tailored behavior change intervention to reduce alcohol use during pregnancy. More emphasis should also be given to pregnant women with no ANC visit, lower gravidity, and an illiterate husband, currently working and Christianity followers.

## Background

Alcohol is a psychotropic agent that can have acute and chronic impacts on brain functions [1]. According to a study conducted in the American region, alcohol use continues to be a significant impediment to the achievement of Sustainable Development Goal 3.5 [2]. Alcohol consumption during pregnancy is a significant public health problem. It has several negative effects on maternal and fetal health [3]. To begin with, alcohol crosses the placenta quickly, with fetal blood alcohol levels surpassing maternal levels within 2 h of maternal admission, affecting fetal development immediately [4]. Second, alcohol consumption during pregnancy may have an indirect effect on fetal development by altering the mother-fetus hormonal connections [5]. According to a World Health Organization research on alcohol use, there is no safe level of alcohol consumption during pregnancy. Furthermore, it states that alcohol is the most prevalent teratogen and dangerous chemical and that there is no safe period or amount of alcohol to consume during pregnancy [6]. On the report of the World Health Organization (WHO), sub-Saharan Africa (SSA) has one of the highest per-capita rates of alcohol consumption in the world, implying a high prevalence of Fetal Alcohol Spectrum Disorder (FASD) in the region [6, 7]. Drinking alcohol is known to cause preventable cognitive impairment in both the child and the mother [8, 9]. One of the few preventable and modifiable risk factors for poor pregnancy and birth outcomes is alcohol consumption during pregnancy [10]. Miscarriage, stillbirth, early birth, congenital abnormalities, intrauterine growth retardation, and low birth weight are all possible side effects [11]. FASD is the most serious condition caused by excessive alcohol drinking during pregnancy [12]. FASD is a catch-all name for a variety of negative consequences on the developing baby caused by alcohol consumption during pregnancy [13]. It includes atypical facial features known as the philtrum, a small head circumference, lower than usual height, low body weight, poor coordination, and Attention Deficit Hyperactivity Disorder (ADHD) [14].

As stated by a paper published in the Lancet Global Health, the global prevalence of alcohol consumption during pregnancy and the occurrence of fetal alcohol spectrum disease is 9.8% and 14.6 cases per 10,000 people, respectively [15].

Women in Chad, Namibia, Uganda, and Ethiopia are the world's biggest alcohol drinkers, consuming 17.7 to 24.5 L of pure alcohol per capita per year [16]. The prevalence of alcohol drinking during pregnancy varies from 2.5% [15] to 59.28% [17], according to researchers conducted in Sub-Saharan African nations. Self-reported alcohol use during pregnancy was found to be 18.5 percent in a study done in Burkina Faso [18]. Unplanned pregnancy [19], a lack of awareness about the effects of alcohol consumption [20], having partners and friends consume alcohol [21], health-related problems such as depression [22] and unemployment [19] are the main factors for alcohol consumption during pregnancy in Sub-Saharan Africa.

Even though alcohol drinking during pregnancy is harmful to both the child and the mother's health, there is a scarcity of evidence in Sub-Saharan Africa. As a result, the goal of this study is to determine the overall prevalence of alcohol intake among pregnant women in Sub-Saharan Africa, as well as its associated characteristics. In addition, the current study is thought to provide crucial evidence for policymakers and program implementers in SSA to avoid alcohol use during pregnancy. Can be utilized as input to strengthen stakeholder and clinical practitioners' efforts to raise awareness among pregnant women about their health outcomes in both the short and long term.

## Methods

### Data source

**Fig. 1:**
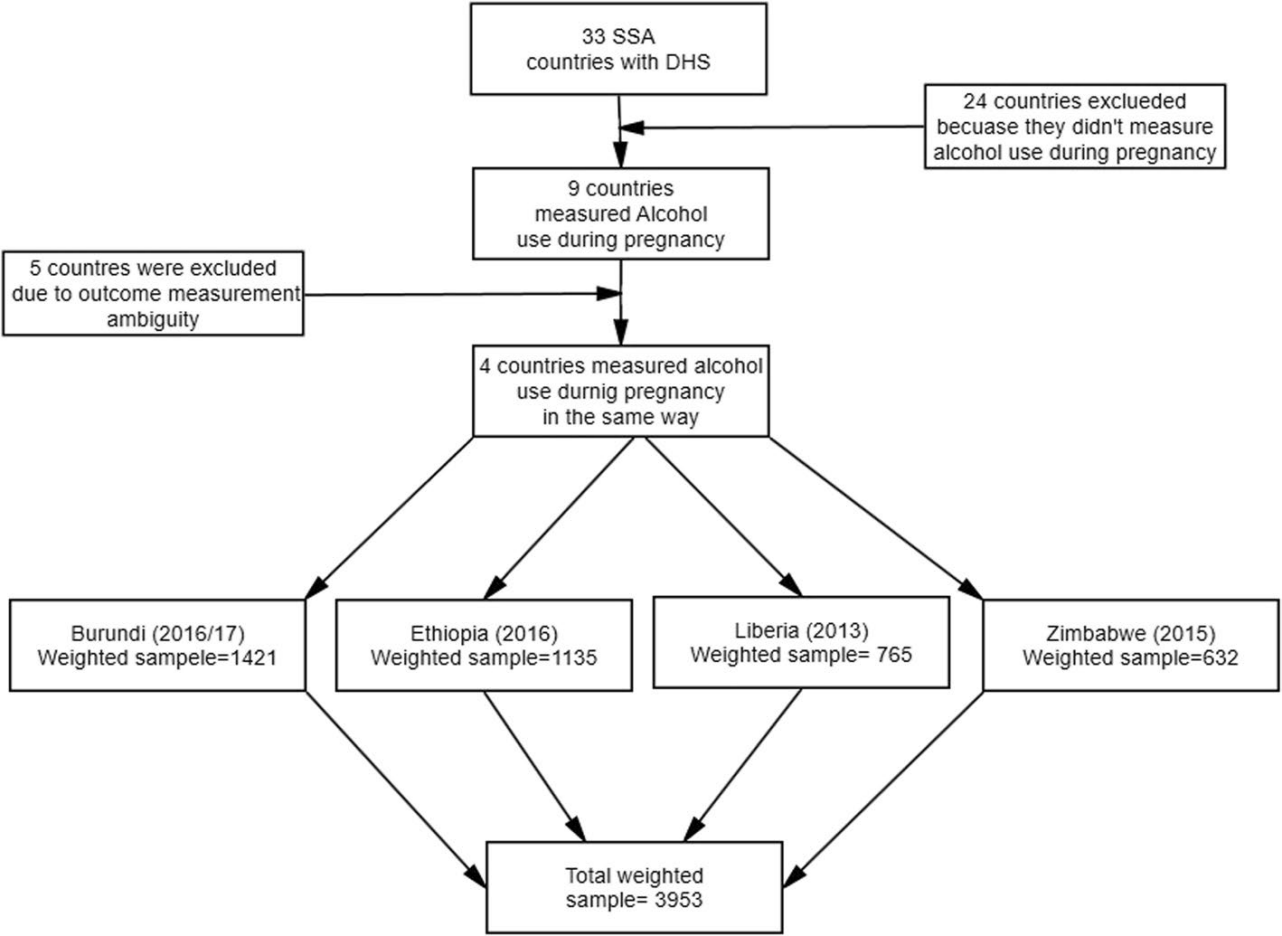
A diagrammatic representation of the sample extracting procedure for the study

This study was based on the most recent Demographic Health Survey (DHS) data from four sub-Saharan African countries: Burundi in 2016–2017, Ethiopia, in 2016, Liberia in 2013, and Zimbabwe in 2015. A total of 33 Demographic and Health Surveys (DHSs) was conducted in SSA from 2010 to 2018. Of these only nine countries were measured alcohol use during pregnancy. However, only four of the nine countries measure alcohol intake during pregnancy in the same way: alcohol intake in the past one month. Therefore, only these four countries were included in the final analysis of the present study, while five of the countries were excluded since they had measured the outcome of interest with no clear/different time frame. Each country’s DHS follows the same execution procedure. A two-stage stratified sampling procedure was used to select study participants in the DHS survey. Initially, Enumerations Areas (EAs) were selected based on the sampling frame of each respective country. In the second stage, a sample of households was selected from each EAs. The detailed sampling procedure used by DHS has been documented elsewhere [23]. Individual records data set (IR file) were used for this study amongst the five DHS datasets. This data set (IR file) is consist of information collected from all eligible women aged 15–49 years. However, this study was limited to women who were pregnant during the survey. Given this, a total weighted sample of 3,953 current pregnant women aged 15–49 years was included in the study from four sub-Sahara African countries (Fig. 1).

## Study variables

### Dependent variable

The outcome variable of this study was alcohol drinking during pregnancy among pregnant mothers in sub-Saharan Africa. The variable was dichotomized into **1** = “drink alcohol during pregnancy” and **0** = “didn’t drink alcohol during pregnancy”.

### Independent variables

In this study, the independent variables included: sociodemographic factors **(**age, religion, residence, marital status, educational status of the mother, educational status of husband/partner, and current working (employment) status, and obstetric factors**:** complications during previous pregnancies (terminated pregnancy), pregnancy plan, and gravidity (number of pregnancy).

### Operational definitions

#### Alcohol drinking during pregnancy

This was defined as consumption of any alcohol-containing drink during pregnancy. It was assessed by asking pregnant mothers whether they took alcohol in the past month.

#### Household wealth quintile

The wealth index was divided into quintiles: poorest, poor, middle, rich, and richest. Principal component analysis were used to arrive at these results (PCA). By combining the lower two (poorest and poor) quantiles with the top two (richest and rich) quantiles, this variable was further divided into three categories (Poor, Medium, and rich) [24].

#### Media exposure

This variable was computed from the frequency of exposure to the two commonest mass media routes (radio and television). In this study exposure to magazines/newspapers was excluded because little (< 5%) women were exposed to this channel. The variable was categorized into two parts: no exposure to media and had exposure to media.

### Data processing and analysis

Individual records (IR) files were used to extract data, which was then coded and transformed using STATA version 14 statistical software. To account for the differential chance of selection and non-response in the original survey, weighted samples were employed for analysis. The presence of statistical significance was determined using multivariable logistic regression analysis. It was fitted after the model's fitness was evaluated using the Hosmer and Lemeshow goodness of fit test. The variance inflation factor (VIF) was also used to analyze multicollinearity across the explanatory components, and it was found to be within acceptable limits. A *p*-value less than 0.05 is used to evaluate the presence of a meaningful effect or relationship of independent factors with the outcome variable.

## Result

A total of 3953 pregnant mothers were included in the study. The mean age of the participants was 27.3 (± 6.8) years with an age range of 15–49 years. The majority (67.9%) of the mothers were Christians and more than half of them (71%) were married and 2938 (75%) were from a rural area. Concerning the reproductive history, about 90.2% (3565) had wanted pregnancy, and (55.7%) of pregnant mothers have ANC visits (Table 1).

**Table 1:** Sociodemographic and obstetric characteristics of pregnant women in sub-Saharan African countries (*n* = 3,953)

Variable	Category	Frequency	Percent
Age (in years)	15–24	1445	36.6
	25–34	1861	47.0
	35–49	647	16.4
Marital status	Single	1145	29.0
	Married	2808	71.0
Residence	Rural	3039	77.0
	Urban	914	23.0
Educational status of respondents	No formal education	1509	38.2
	Primary	1487	37.6
	Secondary	850	21.5
	Higher	107	2.7
Educational status of the husband/partner	No formal education	1097	28.0
	Primary	1349	34.0
	Secondary	964	24.3
	Higher	179	4.5
	Don’t know	364	9.2
Wealth index	Poor	1706	43.0
	Medium	785	20.0
	Rich	1462	37.0
Current working status	Currently working	2099	53.1
	Currently not working	1854	46.9
Mass media exposure	Have exposure	1251	31.7
	Haven’t exposure	2702	68.3
Current pregnancy wanted	Wanted	3565	90.2
	Unwanted	388	9.8
Gravidity ^c^	3 (2–5)^a^		
Have ANC visit	Yes	2201	55.7
	No	1752	44.3
Ever had terminated pregnancy	Yes	625	15.8
	No	3328	84.2

Key: ^a^(median with interquartile range)

### Alcohol use during pregnancy

**Fig. 2:**
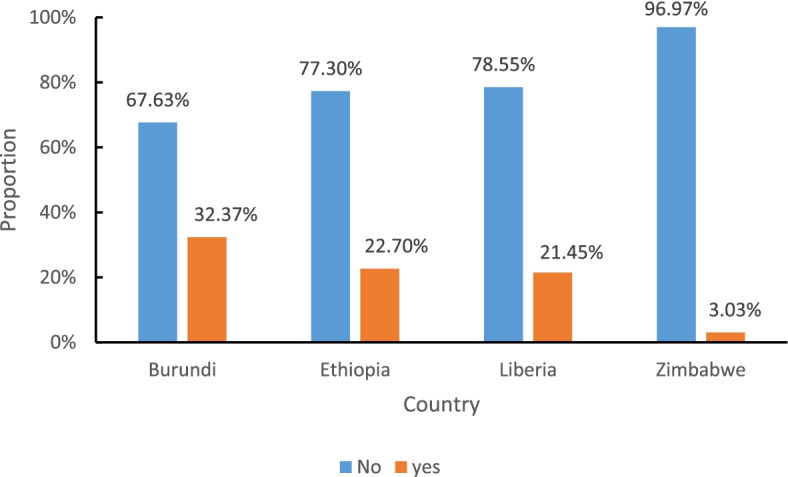
Prevalence of alcohol use during pregnancy among pregnant mothers in Sub-Saharan African countries, based on the recent DHS data (*n* = 3,953)

In this study, the overall prevalence of alcohol drinking during pregnancy was 22.8% with (95% CI (21.5, 24)). Without taking into account population weighting, the prevalence of alcohol drinking during pregnancy for all four countries was 21.7%. The prevalence of alcohol consumption among pregnant mothers was high in Burundi (32.4%) and low in Zimbabwe (3%%) (Fig. 2).

The proportion of alcohol drinking was higher among mothers who are residents of rural areas which is 24% as compared to urban resident mothers (18%). Moreover, the proportion of mothers who drink alcohol during pregnancy is higher among older age groups (35–49) years. Additionally, the proportion of alcohol drinking is higher (27.4%) among mothers who hadn’t formal educational attainment as compared to mothers who were taking part in formal education. In addition to the aforementioned factors, the proportion of pregnant mothers who drank alcohol was higher among single mothers as compared to married ones.

### Factors associated with alcohol drinking during pregnancy

Based on the output of multivariable binary logistic regression analysis the following explanatory variables had a statistically significant association with alcohol drinking during pregnancy: increased age (AOR = 1.02, 95% CI (1.01, 1.04)), being Muslim religion follower (AOR = 0.07, 95% CI (0.05, 0.11)), Husband education (husbands who attended primary educational level (AOR = 0.7,95% CI (0.55, 0.84), have secondary level educational status (AOR = 0.53, 95% CI ( 0.41, 0.7)), husbands who attended higher educational levels (AOR = 0.49, 95% CI (0.31, 0.8). Plus to the above factors being currently working (AOR = 1.3, 95% CI (1.08, 1.55)), gravidity (AOR = 0.92, 95% CI (0.87, 0.99), and having ANC visit (AOR = 0.52, 95% CI (0.68, 0.98) were significantly associated with mothers alcohol drinking during pregnancy.

The odds of drinking alcohol during pregnancy was increased by 2% as the age of the mother increased by a year. The odds of drinking alcohol during pregnancy were 97% lower among Muslim pregnant mothers as compared to Christian pregnant mothers. Moreover, having a husband with primary, secondary, and higher education levels decreased the odds of drinking alcohol during pregnancy by 30%, 47%, and 51% respectively when compared to pregnant mothers having an illiterate husband. (Table 2)

**Table 2:** Factors associated with alcohol use during pregnancy among pregnant mothers in sub-Saharan African countries, (*n* = 3,953)

Variable	Alcohol drinking during pregnancy		*p*- value	AOR
	Yes (*n* = 901)	No(*n* = 3,052)		
	Frequency (%)	Frequency (%)		
Age^a^	28(23–33)^b^	26(22–32)^b^	0.014	1.02(1.05,1.04)
Marital status				
Single	294(25.7%)	851(74.3%)		1
Married	606(21.6%)	2201(78.4%)	0.49	1.08(0.9,1.3)
Residence				
Urban	167(18.3%)	747(81.7%)		1
Rural	734(24%)	2305(76%)	0.19	1.17(0.92,1.5)
Religion				
Christian	848(30.5%)	1935(69.5%)		1
Muslim	22(3%)	694(97%)	0.00	0.07(0.05,0.11)
Other	31(6.8%)	423(93.2%)	0.00	0.24(0.16,0.35)
Mothers educational level				
No formal education	414(27%)	1,095(73%)		1
Primary	354(23.8%)	1,133(76.2%)	0.49	1.07(0.9,1.3)
Secondary	116(13.7%)	733(86.3%)	0.62	0.92(0.69,1.24)
Higher	17(15.8%)	90(84.2%)	0.91	1.03(0.57,1.9)
Current working status				
Currently working	581(28%)	1519(72%)	o.oo4	1.3(1.09,1.55)
Currently not working	320(17.3%)	1533(82.7%)		1
Husband education				
No formal education	336(30.6%)	761(69.4%)		1
Primary education	305(22.6%)	1,044(77.4%)	0.00	0.7(0.55,0.84)
Secondary education	136(14%)	828(86%)	0.00	0.53(0.41,0.69)
Higher	31(17%)	149(83%)	0.006	0.5(0.3,0.82)
Don’t know	93(25.6%)	271(74.4%)	0.03	0.68(0.47,0.97)
Wealth index				
Poor	403(23.6%)	1302(76.4%)		1
Medium	191(24.3%)	595(75.7%)	0.32	1.1(0.9,1.4)
Rich	307(21%)	1155(79%)	0.62	1.06(0.85,1.3)
Media exposure				
Exposed	666(24.7%)	2036(75.3%)	0.85	0.98(0.8,1.2)
Non exposed	235(19%)	1016(81%)		1
Current pregnancy				
Wanted	804(22.6)%	2761(77.5%)	0.75	0.95(0.7,1.3)
Unwanted	97(25%)	291(75%)		1
Have ANC visits				
Yes	527(24%)	1674(76%)	0.03	0.82(0.68,0.98)
No	373(21.3%)	1378(78.7%)		1
Ever had terminated pregnancy				
Yes	165(26.5%)	460(73.5%)	0.17	1.17(0.93,1.5)
No	735(22.1%)	2592(77.9%)		1
Gravidity^a^	3(2–5)^c^	3(2–5)^c^	0.03	0.93(0.86,0.99)

Key: ^a^( continuous),^b^(mean with standard deviation,^c^( Median with interquartile range)

## Discussion

This study assessed the prevalence and associated factors of alcohol drinking during pregnancy among pregnant women in sub-Saharan Africa by analyzing the recent DHS data of the eligible 4 countries in the region. The overall prevalence of alcohol drinking during pregnancy is 22.8 with 95% CI (21.5, 24). Increased age, being a Muslim religion follower, husband/partener educational level, a woman's current working status, having ANC visits, and gravidity of a pregnant woman were factors that were significantly associated with pregnant mothers alcohol drinking.

The result of the present study is in line with the study conducted in Northern Uganda (23.6%) [25]. The possible explanation for this alignment might be the presence of similar socio-demographic characteristics of the respondents. However, the result of the present study is lower than a study conducted in the UK (28.5%), Russia (26.5%) [26], and Nigeria(59.29%) [17]. The possible explanation for the disparity is that media exposure influences social norms about alcohol through advertising, product placements, and stories in a variety of sources, including movies, television, social media, and other forms of entertainment in Western nations [27]. Furthermore, because Nigeria has few alcohol-related policies, ignorance, societal tolerance, and unlimited access to free alcoholic beverages, the prevalence of alcohol consumption among pregnant women in those countries may be higher [17, 28]. On the other hand, the result of the current study is higher than contemporary meta-analysis in sub-Saharan Africa (20.8%) [29], the World Health Organization Africa region (18.5% [16], Korea (16.4%) [30], and Burkina Faso (18.5%) [18]. The disparity could be attributed to differences in study design and variations in the type of measurement tool used to assess alcohol use. That is the study conducted in Korea, which employs the AUDIT (Alcohol Use Disorder Identification Test), as well as a study conducted in Burkina Faso using randomized control trials [18, 30].

Alcohol drinking during pregnancy can also be affected by the different socio-demographic and obstetric characteristics of the mother. In the current study, alcohol drinking during pregnancy was significantly associated with increased age, which is supported by a study conducted in Sweden [31], Uganda [21], and Tanzania [32]. According to the current study's findings, the prevalence of alcohol consumption during pregnancy has increased among Christians, and being Muslim protects against alcohol consumption. This result is consistent with the findings of the Tanzania study [32]. This implied that Women whose religion explicitly prohibited alcohol consumption have a decreased probability of consuming alcohol consumption and concomitantly minimize the chance of alcohol drinking when they became pregnant. Moreover, the current working status of a mother is also significantly associated with alcohol drinking during pregnancy which is supported by a study conducted in Uganda [21] and Zambia [33]. This implied that as the mother is involved in work (employed) she can able to generate income and the probability of buying and drinking alcoholic beverages is concomitantly increased. The educational status of the husband was also another factor that is significantly associated with alcohol drinking during pregnancy, which is supported by a study conducted in Gondar town, Ethiopia [34]. The implies for this association is that husbands' educational status plays a significant role in decision-making power and had a substantially greater impact on maternal health service decisions in developing countries than wives' education level [35, 36]. Consequently, husbands with high educational status might have sufficient knowledge on adverse effects of alcohol use during pregnancy on maternal and fetal health. The result of the present study also revealed that pregnant mothers who had anti-natal care (ANC) visits have a decreased risk for consuming alcohol during pregnancy than their counterparts. And this finding is held up by the study done in Zambia [33]. Finally, the present study also declared the presence of a statistically significant association between alcohol drinking during pregnancy and womens’ number of pregnancy (gravidity). This may be explained as a number of pregnancies increased the chance of the mother to visit health institutions might be increased so the mother had a greater chance to get information about the effect of alcohol drinking on the fetus and in general on pregnancy outcomes.

One of the study's strengths is the use of a large sample size. The potential for deviations from the actual population decreases as the sample gets closer to the actual population. Furthermore, sample weighing was used to overcome disproportionate sample allocations and non-responses.

Because the data is cross-sectional, it may be impossible to establish causal relationships. Furthermore, because the study was based on secondary data, important variables such as the amount, type, and frequency of alcohol consumption among pregnant women were not evaluated. Furthermore, the data set does not include what potential complications a pregnant woman may face during the pregnancy, fetal development, delivery, and neonatal period if she consumes alcohol while pregnant.

## Conclusion and recommendations

The present study revealed that the prevalence of alcohol consumption during pregnancy is high among women’ living in Sub-Saharan Africa. Maternal age, religion, husbands’ level of education, current working status of the mother, presence of ANC visits, and increased maternal gravidity are factors significantly associated with mothers’ alcohol drinking during pregnancy. Thus, Health education programs should be designed to minimize the consumption of alcohol during pregnancy to overcome the unfavorable outcomes that are caused by alcohol consumption on pregnancy and fetal health.

## Data Availability

All result-based data are available within the manuscript and anyone can access the data set online from www.measuredhs.com

## Abbreviations

ANC: Antenatal care
DHS: Demographic and Health Survey
AOR: Adjusted Odds Ratio
FASD: Fetal Alcohol Spectrum Disorder
